# Methylation Assessment of Two *DKK2* and *DKK4* Genes in Oral Squamous Cell Carcinoma Patients

**DOI:** 10.18502/ijph.v49i10.4698

**Published:** 2020-10

**Authors:** Sedigheh KHEIRANDISH, Nosratollah ESHGHYAR, Farzad YAZDANI, Pouyan AMINI SHAKIB, Ali HOSSEINI-BERESHNEH, Zahra NOURI, Ali KHEIRAN-DISH, Fatemeh KARAMI

**Affiliations:** 1. Department of Oral and Maxillofacial Pathology, School of Dentistry, Bushehr University of Medical Sciences, Bushehr, Iran; 2. Department of Oral and Maxillofacial Pathology, School of Dentistry, Tehran University of Medical Sciences, Tehran, Iran; 3. Department of Otorhinolaryngology, Amir A’lam Hospital, Tehran University of Medical Sciences, Tehran, Iran; 4. Department of Medical Genetics, School of Medical Sciences, Tarbiat Modares University, Tehran, Iran; 5. Department of Medical Biotechnology, School of Advanced Technologies in Medicine, Tehran University of Medical Sciences, Tehran, Iran; 6. Department of Medical Genetics, Applied Biophotonics Research Center, Science and Research Branch, Islamic Azad University, Tehran, Iran

**Keywords:** Methylation, Oral squamous cell carcinoma, Methylation-specific polymerase chain reaction

## Abstract

**Background:**

Oral squamous cell carcinoma (OSCC) is one of the most important types of oral malignancies. *DKK* gene family members as well as *DKK2/4* have critical roles in regulation of Wnt signaling as one of the main determining pathway in oral carcinogenesis. This study aimed to identify promoter methylation status of *DKK2/4* genes to provide possible biomarkers for early detection and treatment of OSCC patients.

**Methods:**

A case control study was performed on 31 fresh tissues obtained from oral cavity of patients affected by OSCC and 31 fresh corresponding tissues from normal healthy controls in Tehran and, between the years of 2016–2018. Purified DNA from tissue samples was subjected to bisulfite treatment and then methylation specific polymerase chain reaction (MSP-PCR) was carried out on treated DNA samples.

**Results:**

*DKK4* promoter was methylated in none of OSCC samples while it was methylated in 16.1% of healthy controls. 16.1% of OSCC samples were detected to be semimethylated and 22.6% of healthy normal samples were methylated for *DKK2* promoter gene. Meaningful difference was found in *DKK4* promoter methylation among OSCC patients and healthy controls. Significant correlation was found between *DKK4* promoter methylation and tumor grade. The age of all enrolled samples was demonstrated to have strong effect on promoter methylation of studied genes.

**Conclusion:**

Hypomethylation of *DKK2* and *DKK4* genes in higher grades of OSCC samples may indicate the pivotal role of their expression in tumor cells invasion and progression through modulation of Wnt signaling pathway. Further study required to determine simultaneous expression of those genes and Wnt signaling elements at mRNA and protein levels.

## Introduction

Oral squamous cell carcinoma (OSCC) is the most frequent type of oral cancer and includes the major classification of head and neck squamous cell cancer (HNSCC) with relatively low survival rate and poor prognosis, The rate of OSCC was higher in males and it has higher incidence in South-Central Asia, Central and Eastern Europe, One of the amazing features of OSCC is that both endogenous and exogenous environmental factors have crucial roles in carcinogenesis process. Smoking and drinking alcohol and become infected with Epstein–Barr virus (EBV) and human papillomavirus (HPV) are most important external risk factors of OSCC, Defining the mechanism behind the interaction between environmental and endogenous risk factors not only could pave the way toward targeted prevention and treatment of OSCC but also provide a very earlier screening tests in high risk groups. In this way, determining the genetic and epigenetic profile of tumor tissues in different stages and grade can help to describe the effect of environmental factors on carcinogenesis process.

DNA methylation is one of the main mechanism of epigenetics which can occur within the promoter or body of genes and thereby play a critical role in regulation of gene transcription and expression, 5-methylcytosine which is the most frequent product of DNA methylation process can be usually detected within the cytosine–guanosine dinucleotides (CpGs) lied in GC rich genomic regions called as CpG islands. CpG islands can be mostly found within or next to the promoter sequence and their methylation status can indirectly affect the transcription efficacy. CpG islands hypermethylation is a main cellular defense mechanism against cancer through stabilization of oncogenes and transposons to be untranscribed and silenced, In the other hand, unmethylation of genes which are needed to be actively expressed as well as tumor suppressor or germ cells is a typical expected feature of tumor suppressor genes. The reverse pattern of methylation in either oncogenes or tumor suppressor genes may indicate the role of those genes in the carcinogenesis process.

Dickkopf (DKK) is a gene family comprising for members (DKK1-4) which share common conserved cystein rich protein domain. The general role of this glycoprotein family specifically DKK1/2/4 has been determined as inhibitors of Wnt/ β-catenin pathway which has critical role in embryonic development, cell proliferation and migration, However, the activators effect of DKK2 on Wnt signaling has been demonstrated as much as its inhibitory action, Given that Wnt signaling pathway is a double edged sword in cancer progression, the exact role of DKK family in carcinogenesis has been remained unclear, as well. Moreover, some of this family members as well as DKK2 and DKK1 can function independent of β-catenin and enhance apoptosis process, What is interesting about DKK1 and maybe DKK2 is that unlike other Wnt antagonists, DKK1 overexpression has shown to have targeted specific suppressing effect on Wnt signaling pathway, Owing to this specific effect and some reports indicating hypermethylation of other Wnt pathway antagonists as well as secreted frizzled-related protein (sFRP) in other cancers, we aimed to determine the methylation pattern of *DKK2* and *DKK4* promoter genes which their role and action are less clear,

## Materials and Methods

### Sample preparation

Sixty two fresh tissue samples were obtained from oral cavity of 31 healthy and 31 patients affected by OSCC and stored in −80 °C until further assessments. The study was performed in a case control design in Tehran and, between the years of 2016–2018. Sample size was calculated using Minitab sample size calculator software by consideration of α=0.05, β= 0.2, d=0.15 and *P*=0.3 and it was based on previous report, as well, Design of study was as case control in which normal control samples were collected from individuals reffered for cosmetic periodentic surgeries such as increasing the length of dental crown whom had no medical or familial history of cancer and were healthy. Every patients whom undergone either chemotherapy or radiotherapy besides tumour tissues which were related to extra oral or posterior oral cavity tissues were excluded from study. Inclusion criteria were having pathology confirmation of normal and OSCC and age range of 20–90 yr old.

Consent form was filled for all the enrolled patients and healthy controls according to the local Ethics Committee of Tehran University of Medical Sciences, School of Dentistry (Date of Approval: 20.11.2015. Approval number: 29-08-1394) and ethical standards of the Helsinki Declaration, as revised in 2013 (available at http://www.wma.net/en/30publications/10policies/b3/), as well.

### DNA isolation and modification

DNA was purified from all the samples using QIAamp DNA Mini Kit (Qiagen) and then subjected to treatment with DNA bisulfite kit (EZ DNA Methylation-Gold™ Kit, USA) to convert the unmethylated cytosine into uracil within the promoters of two *DKK2* and *DKK4* genes. The purity and quantity of obtained DNAs were examined by NanoDrop ND-1000 spectrophotometer (NanoDrop Technologies, Wilmington, DE). Promoter Methylation Analysis

The modified DNA sequences were amplified using two primer pairs designed for each methylated or unmethylated templates of *DKK2* and *DKK4* genes in methylation specific PCR (MSP). Primer pair sequences were retrieved from previous report and were confirmed on Methprimer website (Table 1).

**Table 1:** Primer pairs sequences of Methylated and unmethylated PCR reactions

***Gene***	**DKK2**	**DKK4**
Unmethylated primer pair sequences	Forward: 5′-TGTTTTTTAGGTATTGTTGTGTTGGTAGT-3′ Reverse: 5′-ATAAAAAATCAAAAAACATCCCCAAACCA-3′	Forward: 5′-AGAAAAAGTAGTGATAAATAGACGACG-3′ Reverse: 5′-CAACACTATACATCACCAAAACAAA-3′
Methylated primer pair sequences	Forward: 5′-TTTTTAGGTATCGTTGCGTTGGTAGC-3′ Reverse: 5′-AAAATCAAAAAACGTCCCCGAACCG-3′	Forward: 5′-AGAAAAAGTAGTGATAAATAGACGACGT-3′ Reverse:5′-CAACACTATACGTCACCAAAACGAA-3′

The PCR reaction for each of methylated or unmethylated PCRs included 10 pmol of each forward and reverse primers specific for methylated or unmethylated sequences, 2.5 µl of 10x buffer, 2 mM of Mgcl2, 0.2 mM of dNTP mixture and 1U of Hot start Taq DNA polymerase (Fermentase) which was added to 100 ng of each treated genomic DNA samples and then adjusted with ddH2O. After initial denaturation at 95 °C for 5 min, forty PCR cycles (95 °C in 30 sec, 30 sec in annealing temperature, 72 °C in 30 sec) were carried out and pursued by final extension at 72 °C for 5 min. The annealing temperature (Ta) was optimized at 58 °C and 51 °C for methylated templates amplification of *DKK2* and *DKK4* promoter genes, respectively. Ta of unmethylated reactions was optimized as 49 °C and 51 °C for amplification of *DKK4* and *DKK2* genes. PCR products were then resolved on polyacrylamide gel (10%) following AgNo3 staining.

### Statistical analysis

Data was statistically analyzed with the SPSS 17.0 (SPSS Inc, IL, USA). Chi-square, Fisher’s exact and t- tests were implicated to explore the correlation among various variants of the patients and controls. In addition, Mann-whitney U test and spearman regression coefficient test were implicated to find the correlation between clinical grades and gene methylation. In all of the statistical analyses, *P*-value less than 0<0.05 was considered as significant.

## Results

**Fig. 1:**
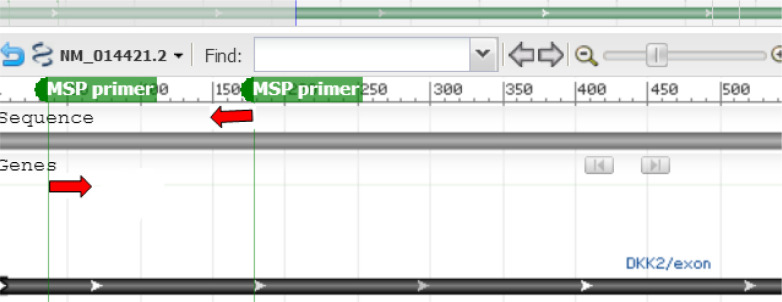
Schematic site of the promoter region of DKK2 gene which has been targeted to be amplified following bisufite treatment

Four primer pairs were used to amplify the modified template sequences of *DKK2* and *DKK4* genes (Fig. 1).

The mean of enrolled patients and healthy controls age were 60.81±15.29 and 38.32±15.25, respectively. Distribution of gender of participants was calculated as 16 male and 15 female among patients and controls.

**Fig. 2:**
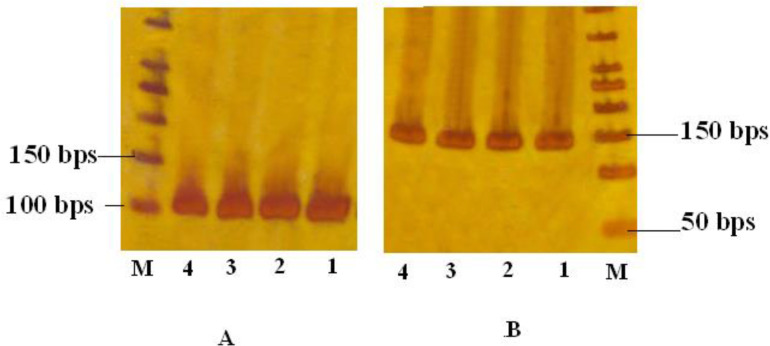
A, MSP-PCR products of regarding unmethylated *DKK4* gene promoter among OSCC patients and healthy controls; B, MSP-PCR products (1–4) of regarding methylated *DKK4* gene promoter among healthy controls. M: Ladder 50 bps.

**Fig. 4:**
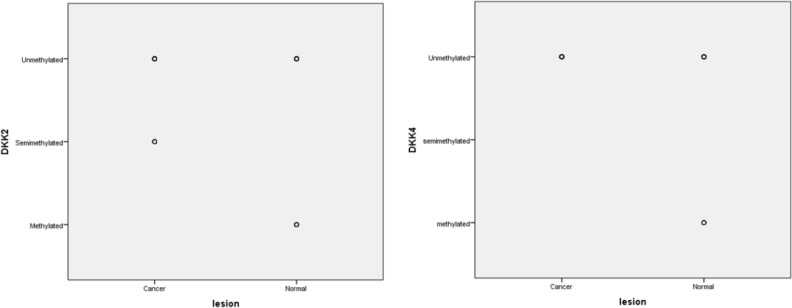
Distribution of methylation status of *DKK2* and *DKK4* genes among cases and controls.

*DKK4* promoter methylation was not detected in no one of OSCC samples while it was methylated in 16.1% of healthy controls (Fig. 2, 4).

**Fig. 3:**
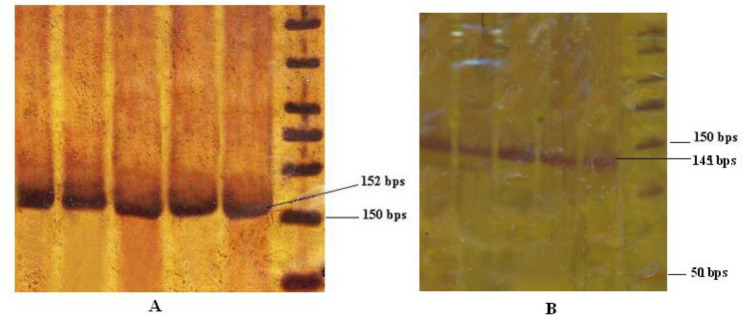
A, MSP-PCR products of regarding unmethylated *DKK2* gene promoter among OSCC patients and healthy controls; B, MSP-PCR products (1–5) of regarding methylated *DKK2* gene promoter among healthy controls. M: Ladder 50 bps

Regarding *DKK2* gene promoter, 5 OSCC samples (16.1%) were detected to be semimethylated and 7 controls (22.6%) had revealed to be methylated (Fig. 3, 4).

Among semimethylated OSCC samples, 3 and 2 tissues were classified as tumor with good and intermediate differentiation, respectively. Mann-Whitney U test analysis have demonstrated that there was meaningful difference in *DKK4* promoter methylation among OSCC patients and healthy controls (*P*=0.02). In contrast of *DKK2* gene, there was significant correlation between *DKK4* promoter methylation and tumor grade (*P*=0.03). Age of enrolled samples including cancer and healthy individuals had strong effect on promoter methylation of *DKK2* and *DKK4* gene promoters (*P*<0.001)

## Discussion

*DKK4* promoter gene was significantly methylated in OSCC samples compared to controls and has shown to be more unmthylated in higher grades. Promoter methylation of *DKK4* and *DKK2* genes was identified in healthy controls whereas methylation status of those genes in OSCC samples was detected as semimehtylation of only *DKK2*. It was semimethylated in 5 OSCC patients with well and moderately differentiated tumor grades while it was unmethylated in 77.4% of the healthy controls. Present failure to detect methylation within the promoter of either *DKK2* or *DKK4* genes in OSCC tissues may be an enough reason on the importance of expression of them in pathogenesis of OSCC. However, in the present study, pattern of demethylation process would be different owing to the identification of semimethylated *DKK2* promoter gene in OSCC cases which was significantly correlated with age. Although it was previously shown to be possible as independent event, hypomethylation of *DKK2* in higher grades of tumor versus semimethylation pattern in low grades may be indicating that overexpression of *DKK2* gene is necessary for tumor transition from low to high grades. Recently, invasion of tumor cells through epithelial mesenchymal transition (EMT) is dependent on a dynamic methylation transition in some genes toward hypomethylation and in some other towards hyper methylation. Herein, although the number of identified samples was low, age dependent methylation of *DKK4* gene in younger healthy individuals would be a further confirmation on this fact that *DKK4* may play as oncogene and should be suppressed in normal non-cancerous status.

Among Dickkopf family, *DKK3* gene and protein expression have been shown to be increased in higher grades of OSCC tumors and head and neck squamous cell carcinoma cells and the cellular localization of protein has been changed, as well. Although the mRNA and protein expression of *DKK2* and *DKK4* genes were not analyzed in the present study, based on the reverse correlation between gene methylation and expression they presumably enroll in the same direction toward OSCC formation and invasion as much as DKK3. However, expression of genes could be regulated independent of methylation status and regardless of unmethylated promoter may be decreased through other mechanism of transcription modulations as well as microRNAs (miRNAs), Overexpression of *DKK2* gene have been found in prostate cancer, medulloblastoma, However, the scenario may be different in higher grades of various other human cancers as well as colorectal cancer, renal cell carcinoma (RCC) and ovarian cancer in which *DKK2* has been shown to be methylated in cancer cell line and identified as tumor suppressor gene, In contrast, *DKK4* gene overexpression has demonstrated in meduloblastoma and its overexpression was found to be correlated with higher rate of invasion and proliferation of colorectal cancer cells which could be in line with promoter hypomethylation of our OSCC samples. However, DKK4 expression was shown to be associated with decrease in hepatoma cells progression induced by thyroid hormones and has been marked as tumor suppressor gene in hepatocellular carcinoma. Based on the aforementioned studies, except of prostate cancer, overexpression pattern of *DKK2* and *DKK4* genes which could be consistent with our finding regarding promoter hypomethylation in higher grades of OSCC tumor tissues may be restricted to head cancers category. In the other hand, defining the exact role of Wnt pathway as proliferative or antiproliferative signaling modules can clarify the possible character of *DKK2* and *DKK4* genes as weather they are tumor suppressor genes or oncogenes in pathogenesis of OSCC.

## Conclusion

Hypomethylation of *DKK2* and *DKK4* genes in higher grades of OSCC tissue samples may indicating their overexpression and thereby tumor progression through suppressing Wnt pathway. By revealing the gene and protein expression pattern of those genes in OSCC samples in further study, the exact role of Wnt signaling would be identified to be as OSCC driver or suppressor. Moreover, finding correlation among *DKK2* promoter methylation, its expression pattern and their correlation with Wnt signaling pathway members are warranted to define the behavior of DKK2 as inducer or inhibitor of Wnt pathway in OSCC pathogenesis.

## Ethical considerations

Ethical issues (Including plagiarism, informed consent, misconduct, data fabrication and/or falsification, double publication and/or submission, redundancy, etc.) have been completely observed by the authors.
